# Persistent left superior vena cava with absent right superior vena cava detected during emergent coronary artery bypass grafting surgery

**DOI:** 10.1186/s40981-015-0004-7

**Published:** 2015-08-27

**Authors:** Yusuke Kusaka, Toshiyuki Sawai, Junko Nakahira, Toshiaki Minami

**Affiliations:** Department of Anesthesiology, Osaka Medical College, Daigakumachi 2-7, Takatsuki, Osaka 569-8686 Japan

**Keywords:** Absent right superior vena cava, Persistent left superior vena cava, Venous malformation, Transesophageal echocardiography

## Abstract

Although persistent left superior vena cava (PLSVC) itself is a common venous anomaly in congenital heart disease, PLSVC with absent right superior vena cava (RSVC) is a rare venous congenital malformation. Due to the lack of symptoms, this malformation is often detected fortuitously when patients undergo central venous catheter placement, pacemaker implantation, or open cardiac surgery. This particular venous malformation is rare, but clinicians in many fields should be well aware of its variations and management techniques to avoid complications. Anesthesiologists should know that patients with PLSVC rarely have absent RSVC. TEE was helpful in the diagnosis of PLSVC with absent RSVC during emergent surgery.

## Background

Although persistent left superior vena cava (PLSVC) itself is a common venous anomaly in congenital heart disease, PLSVC with absent right superior vena cava (RSVC) is a rare venous congenital malformation. Due to the lack of symptoms, this malformation is often detected fortuitously when patients undergo central venous catheter placement, pacemaker implantation, or open cardiac surgery. We present a case of PLSVC with absent RSVC in visceroatrial situs solitus detected by transesophageal echocardiography during emergent coronary artery bypass grafting surgery.

## Case presentation

A 65-year-old man was admitted to our hospital for the treatment of acute myocardial infarction. Electrocardiogram showed significant ST elevation and Q wave but maintained sinus rhythm. Transthoracic echocardiography (TTE) revealed a poor left ventricular (LV) ejection fraction (35 %) with severe hypokinetic LV wall motion. Cardiac catheterization showed severe triple vessel coronary artery disease. During cardiac catheterization, acute hemodynamic instability suddenly occurred. After tracheal intubation, we administered percutaneous cardiopulmonary support (PCPS) and an intra-aortic balloon pump (IABP). As his chest X-ray showed severe pulmonary edema, we scheduled an emergency coronary artery bypass grafting (CABG) surgery.

**Fig. 1:**
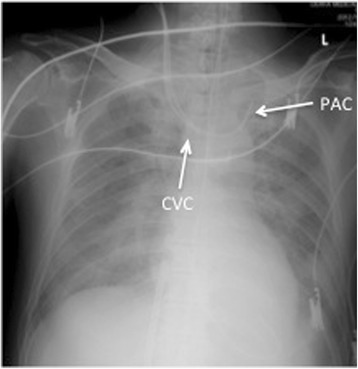
Chest X-ray reveals misplacement of the central venous catheter (*CVC*) and pulmonary artery catheter (*PAC*) in the innominate vein. *L* left

After induction of general anesthesia, we attempted to insert a central venous catheter (CVC) and a pulmonary artery catheter (PAC) via the right internal jugular vein (RIJV). Under ultrasound guidance, the CVC was inserted and secured at 13 cm in depth. Before inserting the PAC, we checked the balloon at the end of the PAC for proper inflation. Flotation was attempted using direct pressure monitoring. Despite several trials, we could not advance the PAC even to the right ventricle (RV) and eventually gave up on inserting the PAC into the pulmonary artery (PA). We temporarily placed the PAC approximately 30 cm from the RIJV. No severe hemodynamic instability was noted during this procedure. Chest X-ray demonstrated that both the CVC and the PAC seemed to have been misplaced in the innominate vein (Fig. 1), so we removed the PAC only. We attempted to insert the PAC via the left internal jugular vein (LIJV). However, we could not advance the PAC to the PA even under radioscopy. Based on the abnormal PAC travelling revealed by radioscopy, we suspected PLSVC and decided against PAC insertion from the upper body.

**Fig. 2:**
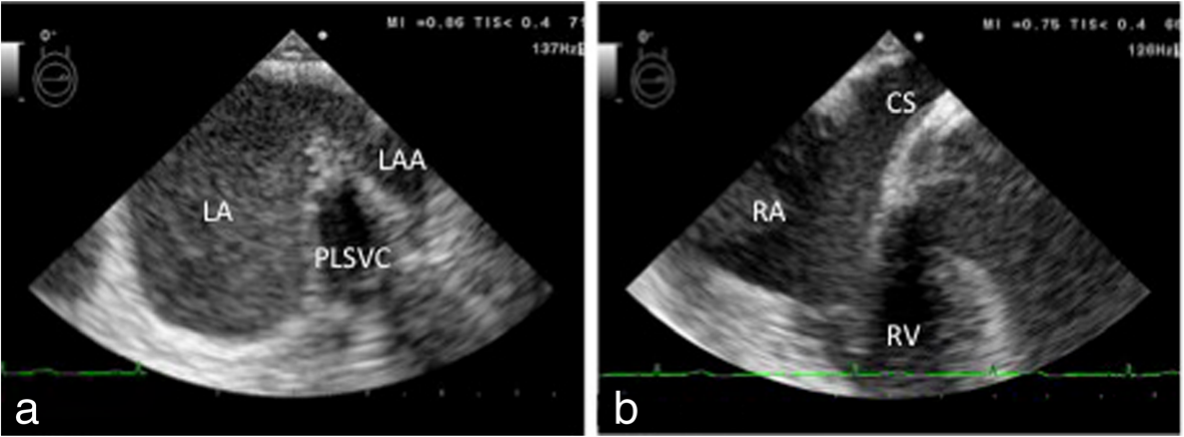
Persistent left superior vena cava (*PLSVC*) was detected in the mid-esophageal aortic valve short-axis view (**a**), and it drained into the dilated coronary sinus (*CS*) in the modified mid-esophageal four-chamber view (**b**). *LA* left atrium, *LAA* left atrial appendage, *RA* right atrium, *RV* right ventricle

**Fig. 3:**
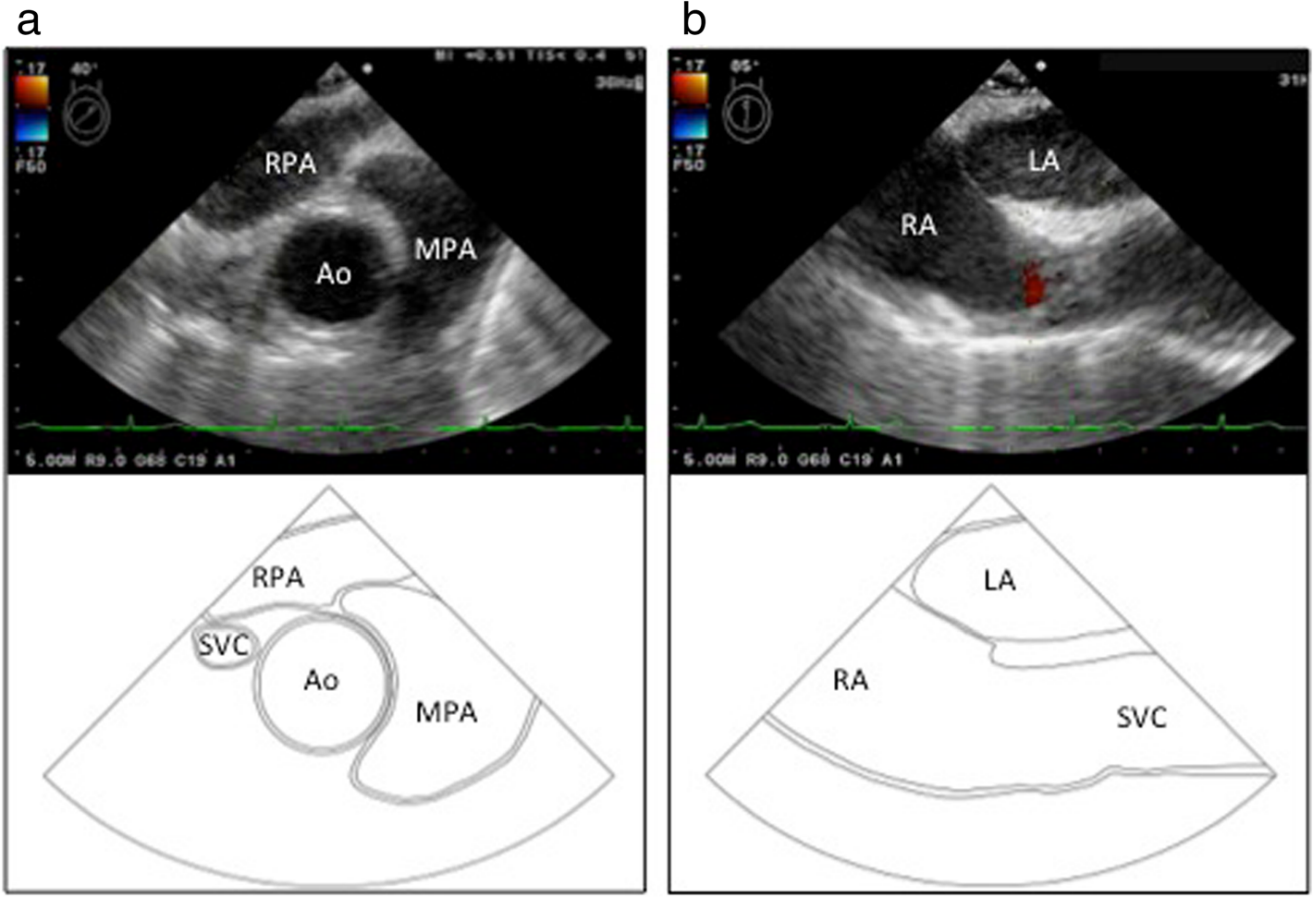
Color Doppler imaging detected no blood flow of the right superior vena cava (RSVC) in the mid-esophageal ascending aorta short-axis view (**a**) and the mid-esophageal bicaval view (**b**). Compare schematic diagrams of normal cardiac structure with transesophageal echocardiography images. *Ao* aorta, *LA* left atrium, *MPA* main pulmonary artery, *RA* right atrium, *RPA* right pulmonary artery, *SVC* superior vena cava

**Fig. 4:**
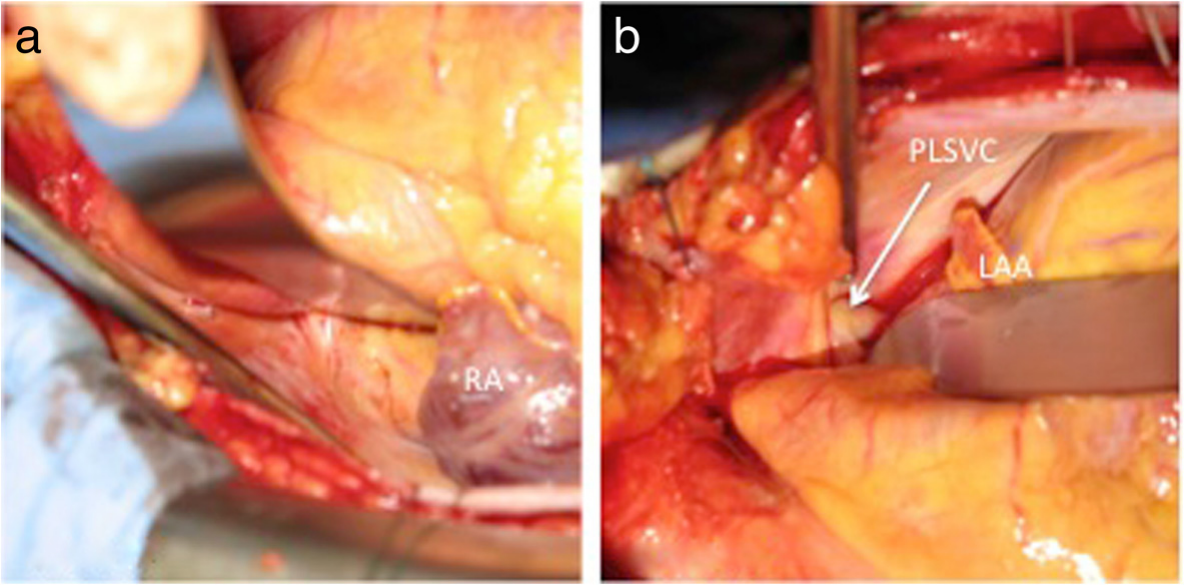
Complete absence of the right superior vena cava (RSVC) (**a**) and persistent left superior vena cava (*PLSVC*) (**b**) were noted. *LAA* left atrial appendage, *RA* right atrium

**Fig. 5:**
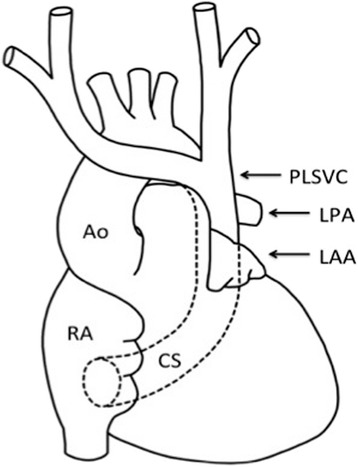
Schematic diagram shows absent right superior vena cava (RSVC) and persistent left superior vena cava (*PLSVC*) draining into the coronary sinus (CS). *Ao* aorta, *LAA* left atrial appendage, *LPA* left pulmonary artery, *RA* right atrium

As expected, intraoperative transesophageal echocardiography (TEE) confirmed the PLSVC, which drained into the dilated coronary sinus (CS) (Fig. 2). The RSVC was not depicted (Fig. 3). From TEE findings, we suspected PLSVC with absent RSVC. Figure 4 shows photographs taken during the intraoperative period. As suspected, the patient had PLSVC with absent RSVC. Intraoperative findings also revealed that both the right jugular vein and subclavian vein drained into the PLSVC through the innominate vein, which is connected to the dilated CS. Figure 5 is a schematic outline of the PLSVC with absent RSVC.

Cardiopulmonary bypass (CPB) was established with ascending aortic cannulation and venous drainage from the right atrium. Conventional CABG was performed on the beating heart. The patient was successfully weaned from CPB with a small dose of inotropes and IABP support. Postoperatively, the patient maintained stable hemodynamics and was transferred to the intensive care unit. On the first postoperative day, the patient was extubated and the IABP was removed. After undergoing a rehabilitation program, the patient was discharged on the 30th postoperative day without complications.

## Discussion

PLSVC occurs in 0.3 % of patients with normal hearts and in 4.5 % of patients with congenital heart disease [1, 2]. PLSVC with absent RSVC (also known as isolated PLSVC) in visceroatrial situs solitus is a very rare venous malformation, occurring in 0.07 to 0.15 % of patients with pacemaker implantations and in 0.09 to 0.13 % of postmortem cases involving congenital heart disease [3–5]. Of those patients with PLSVC, 46 % have other congenital cardiac anomalies, including atrial septal defects, endocardial cushion defects, and tetralogy of Fallot [3].

TEE examinations typically allow for good visualization of RSVC in both the mid-esophageal (ME) bicaval and the ME ascending aorta short-axis views. The combined use of these views is useful for detecting an absent RSVC (Fig. 3). Diagnosis of PLSVC can be confirmed by TEE. In ME views, PLSVC can be depicted close to the left atrial appendage (Fig. 2a). A dilated CS is characteristic of PLSVC and easily visualized in the ME four-chamber view by retroflexing the probe (Fig. 2b). In cases of an absent RSVC, severely increased venous blood flow can result in a giant CS. Enlargement of the CS diameter by more than 1 cm may indicate right atrial hypertension due to anomalous venous return through the CS [6].

In 10 % of PLSVC cases, PLSVC drains into the left atrium (LA) [6]. This causes significant right to left shunt, cyanosis, and paradoxical embolization. Furthermore, drugs can flow directly in the systemic circulation when administered from the left arm. When PLSVC drains into the LA directly, surgical correction should be performed by using an intracardiac baffle [7]. Therefore, when PLSVC is detected by TEE, its entry should be carefully observed. Contrast echocardiography with agitated saline might be helpful for the diagnosis of PLSVC with absent RSVC. Although TEE is very useful for the diagnosis of these venous malformations, it is not so popular in the ordinary clinical setting. Other diagnostic tools such as venous angiography, computed tomography (CT), and magnetic resonance imaging (MRI) could directly visualize these malformations. In the present case, a definitive diagnosis was confirmed with preoperative CT.

PLSVC with absent RSVC itself causes no hemodynamic disturbance [2, 8]. However, various clinical implications are present for establishing CPB and central venous access. In this case, venous drainage for CPB was successful by cannulation into the RA, because bicaval cannulation was not required for the CABG. However, the right-sided open-heart surgical procedure requires venous blood drainage not only from both the superior and inferior vena cava but also from the PLSVC. Direct insertion of the cannula into the CS could result in injury to the CS [9]. The punctuation site for central venous access is a difficult issue. As CS catheterization creates the risk of perforation of the heart, central venous access via the femoral vein might be the safer alternative over that via the SVC [6].

Another issue with abnormal venous drainage is that it sometimes strongly influences the cardiac conduction system. A dilated CS stretches both the atrioventricular node and His-bundle tissue, which can cause tachyarrhythmia and atrioventricular block [6]. Particularly in patients with absent RSVC, sinus node dysfunction occasionally occurs because the early conduction tissue is close to the cardinal venous tissue [4]. Although the patient maintained a sinus rhythm preoperatively, arrhythmias certainly represent an issue that anesthesiologists should carefully note preoperatively.

## Conclusions

Although this particular venous malformation is rare, clinicians in many fields should be well aware of its variations and management techniques to avoid complications. Anesthesiologists should know that patients with PLSVC rarely have absent RSVC. In summary, TEE was helpful in the diagnosis of PLSVC with absent RSVC during an emergent surgery.

## Consent

Written informed consent was obtained from the patient for publication of this case report and any accompanying images. A copy of the written consent is available for review by the Editor-in-Chief of this journal.

## Abbreviations

CABG: coronary artery bypass graft
CPB: cardiopulmonary bypass
CS: coronary sinus
CT: computed tomography
CVC: central venous catheter
IABP: intra-aortic balloon pump
LA: left atrium
LIJV: left internal jugular vein
LV: left ventricle
ME: mid-esophageal
MRI: magnetic resonance imaging
PA: pulmonary artery
PAC: pulmonary artery catheter
PCPS: percutaneous cardiopulmonary support
PLSVC: persistent left superior vena cava
RIJV: right internal jugular vein
RSVC: right superior vena cava
RV: right ventricle
TEE: transesophageal echocardiography
TTE: transthoracic echocardiography
